# Lensless light guide-coupled LED illumination for low-cost microscopy

**DOI:** 10.1016/j.ohx.2025.e00725

**Published:** 2025-11-24

**Authors:** Fox Avery, Thomas Geer, Dirk Albrecht

**Affiliations:** a Department of Biomedical Engineering, Worcester Polytechnic Institute, Worcester, MA, USA; b Nobska Imaging, Inc., Easton, CT, USA; c Departments of Electrical and Computer Engineering and Biology & Biotechnology, Worcester Polytechnic Institute, Worcester, MA, USA

**Keywords:** Microscopy, Optogenetics, Optics, Illumination

## Abstract

Solid-state LED illumination systems for microscopy are generally expensive, often containing multiple LED drivers, excitation filters, and mirrors. High intensity illumination and precise timing are important for *in vivo* and *in vitro* microscopy, fluorescence imaging, and for optical stimulation. Here we present a low-cost, simplified single-wavelength LED mount with 3D printed components that achieves nearly 300 mW optical power through a plastic PMMA light guide, and 40–60 mW excitation intensity with ∼95 % flatfield uniformity at the sample, for under $70 USD. By keeping the LED light beam within the diameter of the light guide, no collecting or collimating lenses are needed. Illumination performance of the low-cost 3 W red and blue systems with passive cooling and plastic light guide match 7 W to 15 W commercial systems with active fan cooling and a liquid-filled light guide, while reducing cost over 20-fold. Additionally, low electrical power use (5 W) and small physical size enable its use in resource-limited environments and compact, battery-powered imaging devices.

## Highlights

• Compact, lens-free illumination system with direct LED-to-light guide coupling.
• High intensity illumination using a 3D printed reflective spacer and plastic light guide.
• 4x higher power efficiency, 20x lower cost than commercial systems.
• Optogenetic stimulation of *C. elegans* elicits equal neural responses vs. commercial LED.

## Graphical abstract

**Figure:**
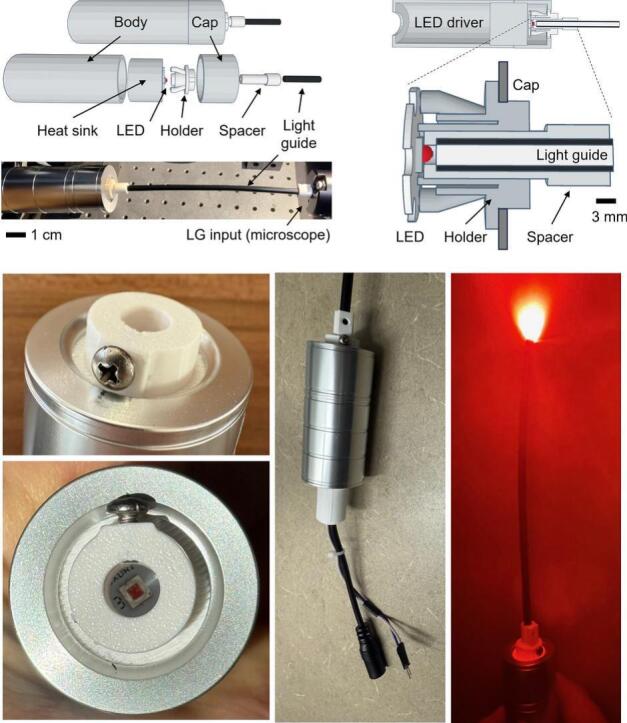

Specifications table

**Table:** 

Hardware name	*Low-cost bright LED*
Subject area	Microscopy, Fluorescence, Neuroscience, Optogenetics
Hardware type	Imaging tools
Closest commercial analog	Mightex GCS-series High-Power Lightguide Coupled LED Source (Type-B, 7 to 15 W) with BLS-series LED controller
Open source license	*CC-BY*
Cost of hardware	*$50*–*70 USD per wavelength*
Source file repository	*Zenodo,**10.5281/zenodo.16944879*

## 1 Hardware in context

Microscopy is a fundamental tool in scientific research that is dependent on quality illumination of samples [[1], [2], [3], [4]]. Illumination sources include incandescent bulbs and solid-state emitters such as lasers and light-emitting diodes (LEDs), which vary in emission spectra, intensity, and illumination uniformity. Fluorescent microscopy in particular relies critically on a bright light source for illumination, as fluorescence emission can be orders of magnitude dimmer than excitation light [5]. Microscopy light sources tend to be expensive, but with growing commercial and industrial development in high power, bright LEDs, such as for home and automotive lighting, low-cost LED modules are now widely available. Here, we present a low-cost illumination system that couples an inexpensive LED module, premounted on a heat sink base, to a standard microscope light guide cable with a unique design that improves overall radiant flux and sample brightness.

Modern scientific illumination systems take advantage of the longevity, color spectrum, and timing control of LEDs. Commercial LED-based systems fall into three primary categories: **broad-spectrum** white light emitters; **narrow-spectrum** single color emitters, and **multi-color** emitters that combine narrow-spectrum emitters with beam splitters and mirrors to merge multiple color beams into a single output (Table 1). Single-color LEDs typically have 20 nm wide spectral bands (FWHM, full-width at half maximum) such that emission filters are still beneficial. Many multicolor systems include filters after each emitter, and most systems cost thousands of dollars (Table 1).

**Table 1:** Solid-state light guide-coupled LED microscope illumination examples. Radiant flux is reported at the LED emitter, at the light guide (LG) output, or at the imaging sample. n.r., not reported.

	**Category**	**Example**	**Radiant Flux** (mW) at LED^, LG, or sample*	**Cost** (USD)
*Commercial*				
	Broad-spectrum (White)	Excelitas, X-Cite XYLIS II Excelitas, Mini+ Lumencor SOLA	∼3000	∼5 k – 8 k
	Narrow-spectrum (Single color)	Mightex, GCS (H-type)	800 – 2600	∼3 k
	Multi-color LED (individually addressable)	Cool LED, pE-4000 Thorlabs Chrolis Lumencor Spectra-X Excelitas X-LED Zeiss Colibri	30* – 250* 40 – 1250 500 ∼500 n.r.	∼12 k – 20 k

*Consumer-built (DIY)*				
	Broad-spectrum (White)	ThorLabs mounted LED	340^– 2500^	∼700
	Narrow-spectrum (Single color)	***This paper*** ThorLabs mounted LED	***290*** 100^– 3000^	***50 – 70*** ∼600
	Multi-color LED (individually addressable)	Angstrom [8]	n.r.	∼130

Illumination systems can be directly mounted to the microscope or coupled via a flexible light guide (LG) cable. Direct-mounted sources provide high brightness illumination, but require microscope-specific mounting adapters and can introduce thermal or electrical interference, vibration from cooling fans, or physical constraints from large heatsinks or Peltier coolers. LGs are advantageous to separate the heat-producing light source from the microscope and come in standardized sizes (3 or 5 mm diameter) to easily couple with different microscopes. In addition, LG-coupled illumination can provide more even sample illumination, as light reflects internally along the LG and emerges with more spatial uniformity. A liquid LG connecting the illuminator to the microscope, typically 1 or 2 m in length, also costs several hundred dollars and may need replacement after several years.

Consumer buildable (Do-it-yourself, or DIY) systems address issues of cost, access, customization, and repairability [6,7]. DIY LED systems have been described for microscope illumination [8,9] and for optogenetic stimulation of mammalian cells, *E. coli* bacteria, *C. elegans* nematodes, or *Drosophila* fruit flies [[10], [11], [12], [13]]. These systems are generally inexpensive and use readily available components, making them accessible to low-resource environments. However, some of these systems require advanced techniques such as custom circuit board fabrication. Many require manual set up for each experiment, resulting in poor light uniformity or repeatability [14], and most do not couple to a light guide. Alternatively, ThorLabs provides individual components that can be assembled into custom illumination systems. Here, our approach is to simplify construction with inexpensive off-the-shelf consumer components and 3D printable parts, couple the LED to a light guide, and prioritize maximizing brightness.

Many specific experiments benefit from bright excitation sources. In neuroscience, optogenetic neural stimulation and recording uses specific wavelengths of light to activate photosensitive proteins and to excite fluorescent reporters of cellular function. For example, genetically-encoded calcium indicators such as GCaMP visualize neuronal activity by increasing fluorescence as intracellular calcium rises in active neurons. Neurons can be activated or suppressed using light-sensitive channelrhodopsin variants such as the red-light activated cation channel Chrimson [15]. Higher excitation light intensity allows for rapid strobing to freeze motion and reduce image blur, stronger photostimulation, and improved signal-to-noise [16]. Here, we demonstrate the performance of the “Low-cost bright LED” system, compared with a commercial system, for rapid photoactivation and neural imaging of sensory neurons in the nematode *C. elegans.*

## 2 Hardware description

### 2.1 Design Rationale

**Fig. 1:**
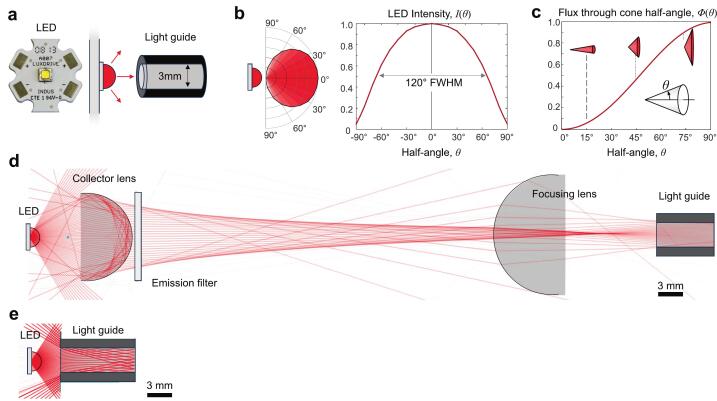
LED to light guide interface. (a) The star base LED module includes a metal heat sink and hemispherical polymer bead focusing lens with a diameter (3 mm) equal to the light guide. (b) Typical LED geometry and beam angle for a 120° full-width half-maximum (FWHM) module, represented in cartesian and polar coordinates. (c) Relative radiant flux through cones of half-angle (θ). (d) A two-lens system uses a collector lens to capture incident light, optionally pass it through an excitation filter, and a collimating lens to focus the wide beam into the narrow light guide. (e) A lensless system brings the light guide close to the LED. For a hemispherical 3 mm diameter LED lens, the maximal light flux into a 3 mm light guide is 47 % of total LED emission in direct contact (θ = 45°) and decreases as LED to LG spacing increases. Adding reflective material around the LED can increase light flux.

The illumination system offers a low-cost, high-intensity, and power-efficient solution that is straightforward to construct and allows for customization. The design directly couples a light-emitting diode (LED) to the light guide (LG), eliminating the need for lenses by keeping light rays within the diameter of the LG. This system uses an off-the-shelf high-power single-emitter 3 W LED on a standard star metal-core printed circuit board (MCPCB) base (Fig. 1a). LED chips typically have a spherical lens cover providing a beam of 120 degrees full-width half-maximum (FWHM, Fig. 1b). The total radiant flux within a narrow cone is relatively small, such that collecting lenses are often used in front of the LED to reflect or refract off-axis light. For example, a lensless system capturing light within ± 45 degrees loses over half of the emitted LED light flux (Fig. 1c).

Typical light-guide coupled LED illumination systems use two lenses, a collector lens creating a wide collimated beam and a focusing lens to refocus the light into the LG with typically 3 mm or 5 mm diameter (Fig. 1d). Multiwavelength systems include emission wavelength filters and dichroic mirrors to combine different colors into a single beam. For single-color systems, the LED and LG can be simply brought in proximity, but efficiency is low as off-axis rays do not enter the light guide (Fig. 1e).

### 2.2 Low-cost LED system with increased efficiency

**Fig. 2:**
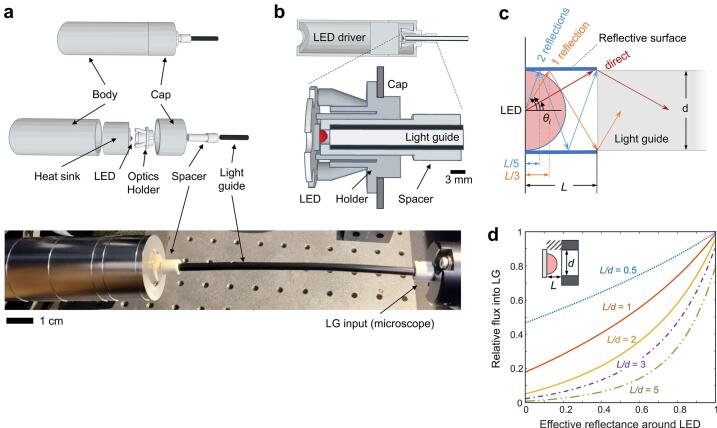
Low-cost, efficient LED system. (a) The LED system is housed in an aluminum cylinder consisting of a hollow body and cap connected by a threaded heat sink attached to the LED module. A 3D printed Optics Holder aligns the LG to the LED via notches in the LED star base. A 3D printed Spacer sets the LG to LED distance. (b) Cross section view shows the proximity of the LED to LG. (c) LED geometry for the radiant flux model. Half-angle θ_i_ represents the maximum at which i reflections occur; for example, from θ = 0 to θ_0_ light rays directly enter the LG. Angles depend on LG diameter d and LED chip to LG distance L. (d) Model output of relative flux into the LG for varying effective reflectance R and geometry L/d. Flux is maximized for shortest LED-to-LLG distance and highest R.

To increase efficiency, reflective material added directly around the LED captures off-angle rays and directs them into the LG (Fig. 2). While LEDs heat during operation (see *Validation and Characterization, 7.3*), the low-cost LED system provides passive cooling to the 3 W LED chip via a large aluminum heatsink and body (Fig. 2a). A two-piece, 3D-printed plastic Optics Holder aligns the LG along six axes, centering it on the 3 mm diameter LED and keeping it perpendicular to the LED axis for optimal light transmission. A white PETG plastic Spacer provides a cylindrical reflective ring around the LED hemispherical lens, directing off-axis light into the LG (Fig. 2, Fig. 2). By keeping the Holder and Spacer separate, it is easy to assemble the metal body, heatsink union and cap during without risk of LED damage (see *Build Instructions*).

By using consumer level LED chips with a passive heatsink, 3D printable adaptors, and no lenses, this system is significantly cheaper and simpler to construct than alternatives (see *Bill of Materials* and *Build Instructions*, below). To further reduce illumination costs, we compared the intensity and flat-field performance of plastic polymethyl methacrylate (PMMA) light guides as an alternative to liquid-filled LGs. In addition to about 50-fold lower cost ($5/m versus $250/m), PMMA can be cut to length allowing for customization and increased light transmission. Optical transmission of PMMA is about 90 % in the visible and infrared light range (400 – 1000 nm), but nearly zero for ultraviolet (UV) wavelengths. The complete low-cost LED system with PMMA light guide costs $50 − $70 USD per wavelength, depending on heatsink size, and requires no advanced manufacturing methods.

### 2.3 Radiant flux model

To estimate the potential gain in illumination brightness by reflecting off-angle light, we modeled the relative flux into the light guide via an effective surface reflectivity, *R*, which includes specular reflectance and diffuse reflectance or scattering. The relative intensity of light at a given angle relative to the LED optical axis, is given by the manufacturer (Fig. 1b), where *θ* is the polar angle from the optical axis. The radiant flux through a cone half-angle is determined by integrating relative intensity over the solid angle defined by the cone, where is the azimuthal angle from the beam axis:

$$\Phi \left(\theta \right)=\int_{0}^{2\pi }\int_{0}^{\theta }I\left(\theta \right)\sin\theta \;d\theta \;d\varphi$$

Radiant flux was calculated using MATLAB R2022a by digitizing the intensity profile, fitting a 4th-order polynomial curve (R^2^ = 0.9997), and numerically integrating for cone half angles from 0 to 90° (Fig. 1c).

For LG diameter *d* and LED-to-LG distance *L*, light within the cone half-angle $\theta_{0}=\tan^{-1}\frac{d}{2L}$ directly enters the light guide. Light within the hollow cone between half-angle $\theta_{i}=\tan^{-1}\frac{d\left(2i+1\right)}{2L}$ and $\theta_{i-1}$ reflects *i* times (Fig. 2c). Luminous flux for each reflection band ($\theta_{i}$ to $\theta_{i-1}$) was calculated for up to 10 reflections. For each band, the relative flux entering the light guide was multiplied by effective reflectivity *R* for each reflection, or *R^i^*. The total flux entering the LG is the sum of all reflection bands (see Fig. 9b, below). For short LG distances, nearly all (>99 %) intensity into the LG comes from direct exposure and the first two reflection bands.

Maximal output brightness occurs with the shortest LED-to-LG distance *L* and highest effective reflectivity *R* (Fig. 2d).

## 3 Design files summary

Design Files are summarized in Table 2.

**Table 2:** List of hardware design files.

**Design file name**	**File type**	**Open source license**	**Location of the file**
P1_Optics_Holder.stl, P2_Spacer_Ferrule.stl, P3_Light_Guide_Ferrule.stl, P4_Cable_Guide.stl	CAD files	CC-BY	*Zenodo,**10.5281/zenodo.16944879*

These design files contain all required parts (P1 – P3) and optional cable guides (P4) in the recommended printing orientations. Files can be imported to any 3D printing slicer software to generate Gcode for any 3D printer.

## 4 Bill of materials summary

The Bill of Materials to construct the Low-cost Bright LED is presented in Table 3.

**Table 3:** List of components with price and references.

**Designator**	**Component**	**Number**	**Cost per unit (USD)**	**Total cost (USD)**	**Source of materials**	**Material type**
E1	Power adapter, 100–240 V AC to 12 V, 2A DC	1	$8.99	$8.99	Amazon https://www.amazon.com/d/B0CMTM6N9M	Electronics
E2	12 V DC pigtail female barrel plug	1	$0.45	$0.45	Amazon https://www.amazon.com/d/B07C7VSRBG	Electronics
E3	2 N3906 PNP transistor	1	$0.07	$0.07	Amazon https://www.amazon.com/d/B01A0QWVHS	Electronics
E4	4.7 K resistor	2	$0.01	$0.02	Amazon https://www.amazon.com/d/B08FD1XVL6	Electronics
E5	22 gage Dupont connector wires	2	$0.07	$0.14	Amazon https://www.amazon.com/d/B07GD2BWPY	Electronics
E6	BNC chassis mount connector	1	$0.99	$0.99	Amazon https://www.amazon.com/d/B087ZK74YB	Electronics
L1-2	Dynamic 10 W LED light module kit *Or:* Dynamic 5 W LED light module kit	1	$51.17 *Or:* $28.30	$51.17 *Or:* $28.30	LEDSupply https://www.ledsupply.com/led-kits/10-watt-led-light-module-kit https://www.ledsupply.com/led-kits/5-watt-led-light-module-kit	Various
L1a	LED housing body	1	Included in kit			Metal
L1b	LED housing cap	1	Included in kit			Metal
L1c	LED heat sink union	1	Included in kit			Metal
L2	Luxdrive 1000 mA BuckPuck (3023-D-E-1000P)	1	Included in kit			Electronics
L3	Cree 1-Up XP-E2 3 W (red, 625 nm or blue, 470 nm, or desired color)	1	$4.99	$4.99	LEDSupply https://www.ledsupply.com/leds/cree-xlamp-xp-e2-color-high-power-led-star	Electronics
L4	LED heatsink adhesive sticker	1	$0.91	$0.91	LEDSupply https://www.ledsupply.com/accessories/hexatherm-tape-thermally-conductive-double-side-tape	Polymer
P1	Optics holder	1	$0.10	$0.10	3D printed, PETG white filament https://www.prusa3d.com/product/prusament-petg-signal-white-1kg/	PETG
P2	Spacer ferrule	1	$0.03	$0.03	3D printed, PETG white	PETG
P3	Light guide ferrule	1	$0.03	$0.03	3D printed, PETG	PETG
P4	Cable guide	1	$0.05	$0.05	3D printed, PETG	PETG
S1	M3 x 6 mm Screws	3	$0.02	$0.06	Amazon https://www.amazon.com/dp/B0CSX4NX7R	Metal
C1	PMMA plastic light guide cable, 3 mm ID	0.2 *m*	$5.25 / m	$1.05	Amazon https://www.amazon.com/d//B0CWGD4LS2	Polymer
			TOTAL:	$68.94 or $46.07		

## 5 Build instructions

### 5.1 LED electrical assembly

The parts needed to complete the LED Electrical Assembly are listed in Table 3, and necessary tools and equipment are listed in Table 4.

**Table 4:** List of electrical assembly equipment.

	Equipment	Model/Type Used	Source
1	Solder	Flux Core Solder, Sn60Pb40	Amazon https://www.amazon.com/dp/B09LDG7J4L
2	Soldering iron	YIHUA 939D + Digital Soldering Station, 75 W	Amazon https://www.amazon.com/d/B07YSCBZ4F
3	Heat shrink tubing	Dual walled, adhesive lined, 3:1 ratio	Amazon https://www.amazon.com/d/B00RC36FWA
4	Heat gun	Mini Hot Air Gun (HG350)	Amazon https://www.amazon.com/d/B08VFY8THD
5	Wire cutter and insulation stripper	6 in. flush pliers	Amazon https://www.amazon.com/d/B097SZRNJV
6	Screwdriver	#1 Phillips	

#### 5.1.1 Solder wires and electrical components for LED control

**Fig. 3:**
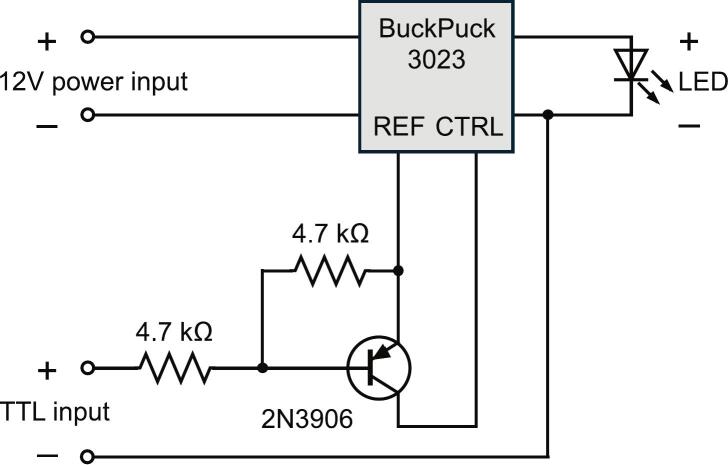
Circuit diagram for LED power and TTL (on–off) control.

The electrical circuit diagram (Fig. 3) allows for digital strobing control of the LED at a maximum frequency of 10 kHz. A TTL “high” (5 V) signal turns on the LED and “low” (0 V) turns it off. While the BuckPuck can deliver a graded output current via an analog voltage at the control (CNTL) wire, the current or intensity scaling is not directly proportional to control voltage. Instead, a full range of graded output intensity can be controlled with this digital control circuit by pulse-width modulation (PWM) from a timer circuit or microcontroller.

**Fig. 4:**
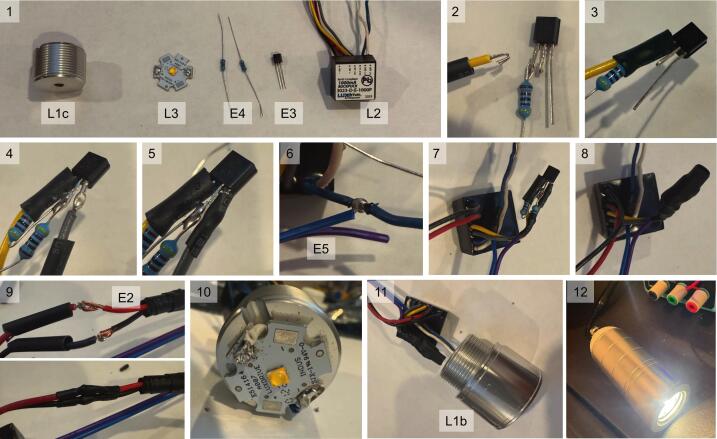
Constructing the LED control circuit. (1) Control circuit components. (2–12) Example fabrication photos. See Table 3 for part information.

Electrical components and suggested circuit construction steps are shown below (Fig. 4). Standard soldering techniques are sufficient to achieve good results, such as the use of Sn60/Pb40 flux core solder and a clean, tinned solder iron set to 330 – 370°C. Ensure minimal heating of the components and consult the LED data sheet for specific thermal limits of the LED while soldering.

On the BuckPuck driver (**L2**), strip the yellow REF wire and the grey CTRL wire by ∼ 5 mm, and solder the REF wire and a 4.7 kΩ resistor (**E4**) to the emitter pin of the 2N3906 transistor (**E3**). Before each solder connection, add heat shrink tubing for insulation (*photo 2*). Make a hook and link each wire for a secure solder connection. After soldering, heat the connection with the heat gun until the tubing is shrunk (*photo 3*). Next, solder the other resistor lead and a second 4.7 kΩ resistor (**E4**) to the base pin of the transistor (*photo 4*). Solder the gray CTRL wire to the collector pin of the transistor (*photo 4*) and insulate with heat shrink tubing (*photo 5*).

Choose an appropriate signal (TTL control) connector pair, such as a Dupont header connector (**E5**) or BNC jack or pigtail (**E6**). Strip ∼ 5 mm off the end of the (−) TTL control wire to the blue LED(−) wire near the BuckPuck (*photo 6*). Strip and solder the (+) TTL control wire to the end of the second resistor (*photo 7)*. Once complete, place a large heatshrink tube over the transistor and resistor assembly (*photo 8*).

**Fig. 5:**
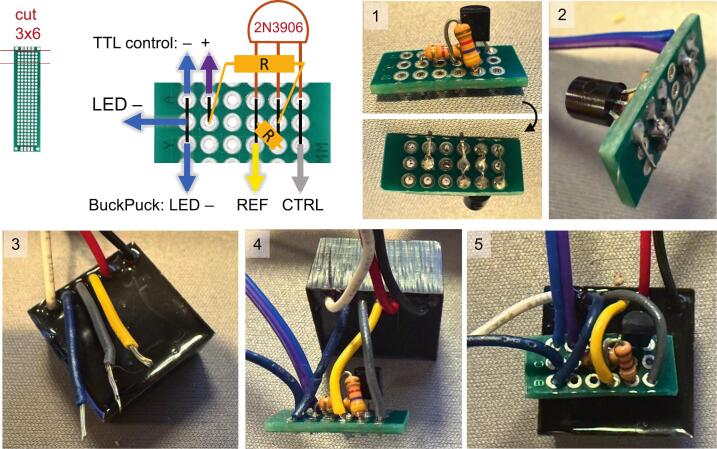
Alternate construction of the LED control circuit. The board layout and example fabrication steps are shown. Black lines on the circuit layout represent solder connections on the bottom of the board.

Alternately, a small perforated circuit board can be used to fabricate the circuit (Fig. 5). A 3 x 6 hole board is conveniently trimmed from a longer board by scoring cut lines with a box cutter, aligning the cut line to a vise jaw edge, and bending the board over the edge until it snaps. Solder the components according to the circuit layout (Fig. 5 and *photo 1*). Then connect the 3 wires from the BuckPuck driver (**L2**), the TTL control wires (**E5** or **E6**), and the LED(−) wire as indicated (*photos 2*–*5*). Ensure proper solder connections along each column on the back of the board (Fig. 5 circuit layout, black lines), with no shorting across columns.

#### 5.1.2 Power connection

Strip ∼ 5 mm off the ends of the 12 V pigtail female cable (**E2**) and solder the red VIN(+) BuckPuck wire to the red wire 12 V pigtail cable and black VIN(−) BuckPuck wire to the black 12 V connector wire (Fig. 4, *photo 9*). Use heat shrink tubing to secure and insulate the wire connections.

#### 5.1.3 LED connection

Strip ∼ 5 mm off the end of the BuckPuck (**L2**) white LED(+) wire and the blue LED(−) wire. Run these wires through the hole in the middle of the threaded heat sink union (**L1c**) so that the grooved cut is on the same side as the end of the wires. Separate the two wires so that they sit in the groove with about 10 mm of wire past the edge of the union. Apply the heatsink adhesive sticker (**L4**) to the backside of the LED chip (**L3**) with firm pressure. Next, line up the LED chip to the grooved surface of the union so that the positive and negative wires can reach the positive and negative terminal pads on the LED chip (Fig. 4, *photo 10*). Remove the sticker backing to reveal the adhesive, center the LED chip, and firmly press it onto the union. Do not touch the actual LED chip. Solder the ends of the two wires to their respective terminals on the LED chip.

### 5.2 Print 3D parts

**Fig. 6:**
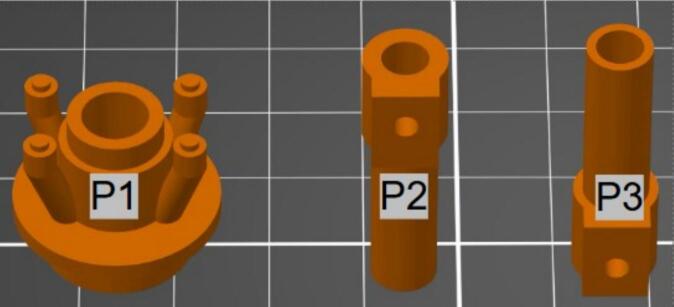
3D printed components. P1, LG optics holder. P2, Spacer Ferrule. P3, LG ferrule. These orientations allow for optimal printing with minimized support and easy support removal.

Download provided STL files in the Design File Summary (Table 2). Import these files into 3D slicing software (such as PrusaSlicer, Cura, etc.) and maintain scaling, orientation, and metric (mm) units. Orient the Optics Holder (**P1**) with the spacer entrance facing the build plate and the 4 alignment legs facing away. Orient the Spacer Ferrule (**P2**) with the set screw hole at the top. Orient the Light Guide Ferrule (**P3**) the opposite way (see Fig. 6, below). Recommended print settings are 0.2 mm layer height, 30 % infill, and organic (tree) supports. Print with white PETG (e.g., Signal White PrusaFilament) or other higher-temperature filament, such as ABS. PLA filament is not recommended, as LED temperature is likely to cause warping. After printing, remove support material and inspect for defects or misprint errors.

### 5.3 LED case assembly

Place the 3D printed Optics Holder (**P1**) onto the LED chip, aligning the 4 legs of the holder with 4 half-circle notches on the side of the star base LED (**L5**). This ensures that the holder is centered on the LED. While pressing the holder down, place the cap (**L1b**) over the holder and thread the cap onto the union until the holder is secure (Fig. 4, *photo 11*). Place the completed electrical circuit assembly, BuckPuck driver, 12 V barrel plug, and TTL wires into the housing body (**L1a**) running the barrel plug and TTL wires through the hole in the back of the case. Thread the housing body onto the union (**L1c**), ensuring that the wires and BuckPuck do not get tangled or twisted, for example by using a screwdriver or rod to prevent the circuit components from rotating.

### 5.4 Prepare PMMA cable

Cut the PMMA light guide cable (**C1**) to the desired length, such as 20 cm. While pressing the cable flat against a cutting surface, use a razor blade to make a smooth, perpendicular cut. Inspect the end of the cable to ensure a uniform PMMA surface, and recut if necessary.

### 5.5 Final assembly

#### 5.5.1 Attach spacer ferrule to LED

**Fig. 7:**
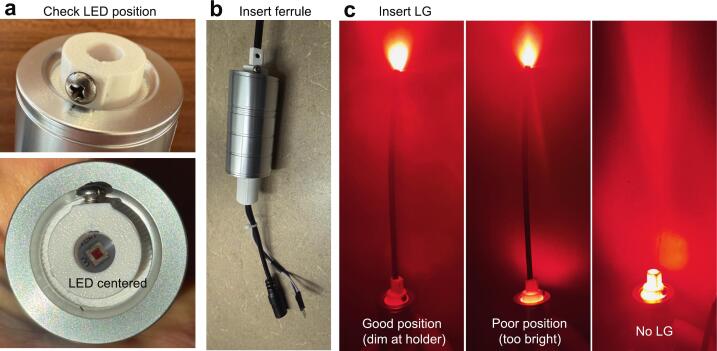
LG and spacer alignment and positioning. (a) With power off, check that the LED is centered in the LG holder. Do not look directly at the LED when powered on. (b) Insert LG spacer ferrule while LED is off and cold, to avoid any deformation of the LED lens. Press until it makes contact with the LED base. Tighten the screw to secure and prevent rotation. (c) Insert the LG fully. Check its position by briefly turning on the LED. The LED holder should be dim (left panel), indicating that most of the light enters the LG. Compare to examples with insufficient insertion (middle) or no LG (right).

Ensure good alignment of the LED in the center of the Optics Holder (**P1**) as in Fig. 7a. Insert the spacer ferrule (**P2**) into the holder until it contacts the LED chip (see Fig. 2b). Use a screw (**S1**) to hold the ferrule in place (Fig. 7b). Ensure this step is performed with the LED off and cold, such that any minor misalignment does not damage the LED lens, which can soften with heat.

#### 5.5.2 Insert LG into LED assembly

Slide the PMMA LG cable (**C1**) (or liquid LG) into the Spacer Ferrule (**P2**) until it contacts the ferrule end stop. This ensures a 1.5 mm gap between the LED base and the LG and direct contact between the hemispherical LED lens (3 mm diameter, 1.5 mm height) and the LG, without applying pressure (see Fig. 2b). Briefly turn on the LED to evaluate its position (Fig. 7c), then use a screwdriver to gently tighten a screw (**S1**) until the cable is secured. A properly positioned LG will show little light through the ferrule (Fig. 7c), indicating most of the light enters the LG.

#### 5.5.3 Attach LG to microscope

Slide the other end of the PMMA LG into the Light Guide ferrule (**P3**) until it is flush with the end of the ferrule, then tighten the screw (**S1**). Insert the LG ferrule into the microscope launch. Adjust the insertion distance to optimize illumination intensity and uniformity (See *Operations* 6.3 and *Characterization and Validations* 7.4). Tighten the microscope launch screw to secure the light guide.

## 6 Operation instructions

### 6.1 Stand and alignment

**Fig. 8:**
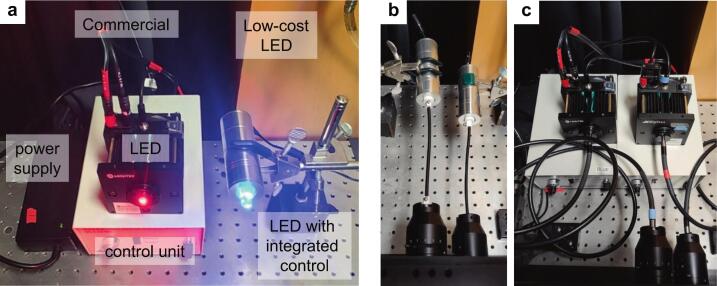
Photos of commercial and low-cost LED systems. (a) Labeled components for each system. The low-cost LED controller is housed within the aluminum body. (b) Example low-cost red and blue LED setup with 20 cm PMMA LGs. (c) Mightex GCS high-power red and blue LEDs mounted to 2 m liquid LGs. (For interpretation of the references to colour in this figure legend, the reader is referred to the web version of this article.)

Mount the LED level with the microscope LG launch, with the LG connecting straight from the LED chip to the microscope light path. For example, use a magnetic base or optical breadboard post to clamp the aluminum LED casing (Fig. 8).

### 6.2 Connect power and control wires

Connect a 12 V power adaptor (**E1**) capable of supplying at least 1A current per LED system to mains power and the LED power input jack. Connect TTL control signal wires to the illumination control source via BNC cable or other wiring. Control signals can come from most cameras via their *Exposure* output signal or a configurable digital output, or from a timing controller box [17], or from microcontroller circuits [18]. Using control software, send a test signal to the light source to verify system power and function.

### 6.3 Optimize sample illumination

The position of the light guide in the microscope launch affects sample illumination intensity and uniformity (see section 7.4 and Fig. 10). To measure intensity and uniformity, place a fluorescent slide (or a glass slide with smooth, bright white paper) on the sample stage. Using microscope control software, turn on live view and change the live image color scaling to best show intensity differences. Adjust the LG insertion distance for optimal image brightness and uniformity across the desired field of view. For more precise analysis, capture images, average them to reduce noise, and measure pixel intensity using the linescan feature.

## 7 Validation and characterization

### 7.1 LED illumination intensity

**Fig. 9:**
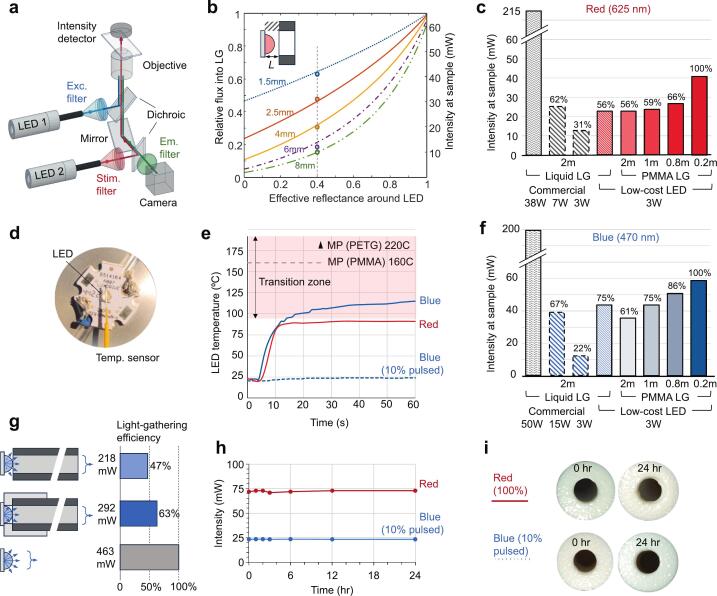
Low-cost, high-power LED system performance. (a) Experimental setup to measure intensity at the microscope sample. (b) Model prediction of relative output flux for varying LED-to-LG distance L and effective reflectance R. Experimental data for white PETG spacers were fit to model curves to estimate R. (c) Light power measurements for different red LED sources (Low-cost LED or commercial) of the indicated power (wattage) and light guides (2 m liquid or 0.2 – 2 m PMMA). Labels indicate relative output power compared to the best low-cost LED configuration. Bars represent mean, n = 3. (d) Experimental setup for measuring LED temperature. (e) LED temperature measured for red (625 nm) and blue (470 nm) LEDs turned on at t = 5 s either continuously or pulsed with a 10 % duty cycle (10 ms pulse at 10 Hz). MP, melting point of PETG or PMMA. In the transition zone, plastic materials can deform. (f) Light power measurements for blue LEDs as in panel c. (g) Light gathering efficiency for configurations with no optics holder or spacer (top), with holder and reflective spacer (middle), and estimated total LED output (bottom). Output was measured with the blue LED and a 0.2 m PMMA LG. (h) Intensity measurements across 24 h for 100 % duty cycle continuous red light and 10 % duty cycle blue light. (i) Images of spacer ferrule before and after 24 h of continuous use. (For interpretation of the references to colour in this figure legend, the reader is referred to the web version of this article.)

We compared radiant flux at the imaging sample for different low-cost LED colors (red and blue, 3 W) and LG spacer geometries. Additionally, two higher power commercial systems, Mightex GCS-0625–38 (red, 38 W) and GCS-0470–50 (blue, 50 W), were measured for comparison (Fig. 8). Light power was measured using a 25.4 mm diameter sensor (Thorlabs S425C) placed at the sample plane (Fig. 9a). The experimental set up consisted of a custom epifluorescence microscope (ASI RAMM) capable of visualizing green emission with a camera (Orca flash 4.0 v2 sCMOS) at a magnification of 4x (Olympus 0.28NA objective lens). Blue excitation light (470 nm) entered the light path through a LG launch on the side and filtered with EGFP excitation (ET470/40x) and emission (ET525/50 m) filters and dichroic mirror (T495lpxr) (Fig. 9a). Red stimulation light (625 nm) entered the light path through a LG launch with a 645/75 excitation filter and 590 SP dichroic mirror. The low-cost or commercial illumination systems were attached to the microscope launches via a 2 m liquid LG or varying lengths of PMMA LG (0.2 – 2 m) cut by a razor blade with no further end polishing (Fig. 8). At least three intensity measurements were made for each setup after disassembling and reassembling the LED system and LG. We also measured LED temperature (Fig. 9, Fig. 9), monitored output intensity over time, and examined the plastic ferrules for deformation after 24 h.

The low-cost red LED with 0.2 m PMMA LG produced a maximum of 41 mW light power at the sample plane with the minimum 1.5 mm spacer. Power reduced with increasing spacer length to 10 mW output with an 8 mm spacer (Fig. 9b). Increasing PMMA length from 0.2 m to 2.0 m decreased output power 44 % to 23 mW, comparable to the 2 m liquid LG (Fig. 9c). The 41 mW maximal output power (with 1.5 mm spacer and 0.2 m PMMA LG) was about four-fold lower than the commercial high-power 38 W system with liquid LG, and used far less electrical power (5 W vs. 120 W) as measured using a power-sensing outlet strip. Thus, the low-cost LED system had a 4-fold better optical-to-electrical power ratio at the sample plane of 8 mW/W (41 mW / 5 W) compared to 2 mW/W (215 mW/120 W) with the commercial system.

Similar measurements for low-cost blue LED with 0.2 m PMMA LG produced a maximum of 59 mW with the 1.5 mm spacer and 9 mW with the 8 mm spacer. Changing the PMMA length from 0.2 m to 2.0 m decreased power by 39 % to 36 mW, similar to the 2 m liquid LG (Fig. 9f). The maximal intensity for the low-cost blue light was approximately 3-fold lower than the commercial high power 50 W system and used far less electrical power (5 W vs. 70 W). The blue low-cost LED had a 4-fold better optical-to-electrical power ratio at the sample plane of 12 mW/W (59 mW/5W) compared to the commercial system of 3 mW/W (200 mW/70 W). Comparisons of the low-cost LED systems and commercial systems were similar for both LED colors.

### 7.2 Efficiency estimation

To estimate light-gathering efficiency, light power was first measured at the end of the 0.2 m PMMA LG manually aligned and directly touching the blue 470 nm LED lens, with no optics holder or reflective spacer (Fig. 9g). The 218 mW measured power represents the 45 degree cone half-angle (*L* = 1.5 mm; *d* = 3 mm, see Fig. 2c) and corresponds to an estimated 47 % of total LED light power (463 mW, see Fig. 1c). Using the white 1.5 mm PETG spacer, output power rose 34 % to 292 mW, or an estimated 63 % of incident power. At the sample, measured optical power of 59 mW (Fig. 9f) suggests an 80 % loss in light through the microscope due to spectral filtering, apertures, and surface losses [19,20].

Estimated effective reflectivity *R* was calculated from the light power ratio for 8 mm vs. 1.5 mm spacers and fit to the model curves (Fig. 2d), with *R* = 0.4 providing the best fit (Fig. 9, Fig. 2, R2 = 0.996). This suggests that improvements to reflectivity of the ring surrounding the LED lens could further increase output light flux, such as by reflective paint or polished metal ferrule.

### 7.3 Heat dissipation

High intensity LEDs generate heat and require a passive heatsink or active fan or thermoelectric cooling. For example, the Mightex 470 nm Type-H high power LED uses a large fan cooled heat sink and draws ∼ 70 W at the power outlet and 50 W at the LED. The low-cost LED draws only 5 W at the power outlet and 3 W at the LED, such that passive heatsink cooling is sufficient.

To evaluate the suitability of 3D print filament and plastic PMMA LG materials in close proximity to the hot LED, the LED chip temperature was measured with a microprobe thermometer (Omega HH801B with Type K thermocouple) (Fig. 9d). The maximum temperature of both 470 nm and 625 nm LEDs were below the melting points of both PETG (160 °C) and PMMA (220 °C) (Fig. 9e). Continuous red 625 nm illumination reached a steady 85 °C at the LED surface, which nears the glass transition temperature for PETG and PMMA. In the thermal transition zone, plastic deformation is possible if force is applied, but the Optics Holder and Spacer design minimizes any force on the plastic closest to the hot LED. Continuous blue 470 nm light exceeded 115 °C, whereas pulsed blue light at 10 % duty cycle (10 ms illumination every 100 ms) remained near room temperature. Pulsed excitation light (for example, restricted to camera exposure time) is preferred for imaging to minimize photobleaching.

Over 24 h, intensity output remained constant (Fig. 9h) and no deformation of PETG or PMMA was observed after continuous red or pulsed blue illumination (Fig. 9i). However, we verified that a spacer printed with a lower melting point filament (poly-lactic acid, PLA) warped and deposited material on the LED.

### 7.4 Illumination field uniformity

Uniform illumination intensity across the microscope field is important for image quality and quantitative measurements, although image postprocessing can correct pixel intensity values affected by uneven illumination. Other applications such as optogenetic activation of neurons require more spatially uniform excitation light to equally stimulate across the optical field and minimize variability in neuronal response.

**Fig. 10:**
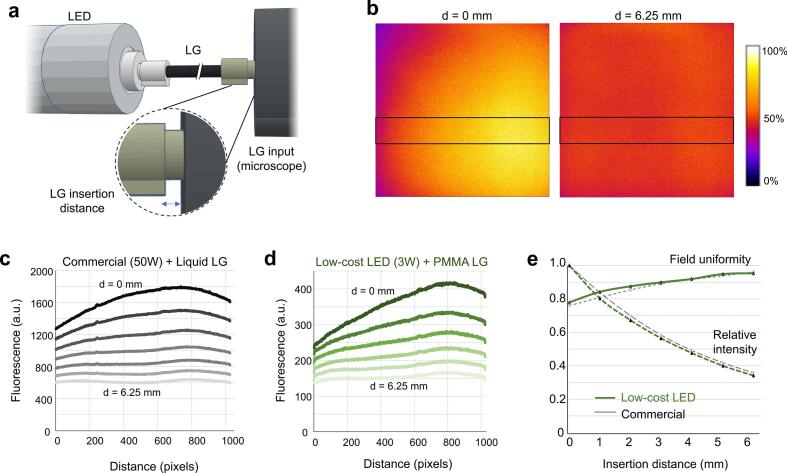
Illumination field uniformity using the low-cost LED and PMMA light guide. (a) Field flatness varies with LG insertion distance in the LG input launch. (b) Flatfield illumination images were obtained varying insertion distance from 0 − 6.25 mm and recording epifluorescence frames using a uniform fluorescent plastic slide (Chroma). Experimental setup is shown in Fig. 9a. Boxed region represents a 100-pixel wide linescan. (c) Linescans show mean pixel values subtracting dark pixel values for the commercial system and varying insertion distance. (d) Linescans of the low-cost LED system as in panel c. (e) Relative peak intensity and field flatness for varying insertion distance. Field flatness is measured from panels c,d as the minimum pixel value divided by the maximum pixel value across the linescan. A field uniformity of 1.0 is ideal.

The LG launch geometry and LG insertion distance affect both field uniformity and overall intensity. We measured illumination uniformity by imaging a fluorescent plastic slide (Chroma 92001) and analyzing pixel intensities using a 100-pixel wide band and the linescan function in ImageJ. Field flatness was quantified as the minimum (edge) intensity divided by the maximum (center) intensity after subtracting the camera dark pixel value, where 1.0 represents perfect field flatness. Relative maximum intensity and field flatness were measured for LG insertion gap distances from 0 to 6.25 mm (Fig. 10a). In this range, increased insertion spacing increased field flatness but decreased maximum intensity (Fig. 10b-d). No differences were observed in relative intensity or field flatness comparing the low-cost LED system with 0.2 m PMMA LG and the commercial system with 2 m liquid LG (Fig. 10e). In this setup, an optimal balance of flatness and intensity occurred at about 3 to 5 mm insertion distance. The optimal position depends on the specific light guide launch and microscope, as well as the experimental application, such as whether brightness or uniformity is more important.

### 7.5 Use case: optogenetic activation and neural imaging in the nematode *C. elegans*

A use case for this illumination system is the optogenetic stimulation of *C. elegans* and optical readout of neural responses [21,22]. To demonstrate performance of the low-cost LED system compared with a commercial system, we recorded optically-activated responses in AWA chemosensory neurons in a microfluidic device, similar to previous reports [23]. Microfluidic arenas allow for control of the local environment and precise delivery of chemical stimuli to animals housed in an optically clear platform that is ideal for quantitative imaging [[23], [24], [25], [26]].

**Fig. 11:**
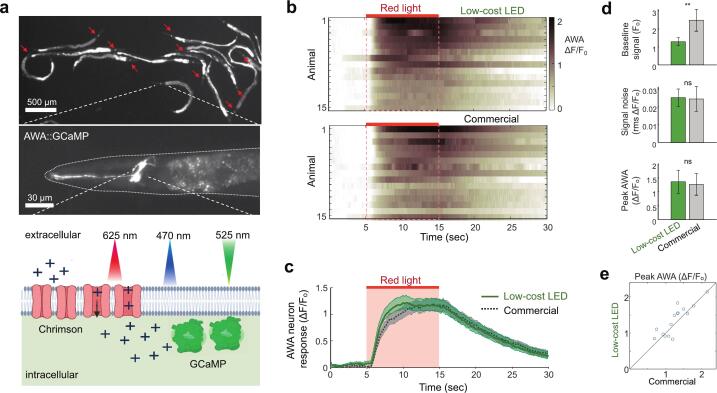
Example neuronal recordings in C. elegans, using the low-cost LED with PMMA LG or commercial LED and liquid LG. (a) Microscope image of C. elegans nematodes expressing the calcium sensor GCaMP and the light-activated cation channel Chrimson in the AWA chemosensory neurons. Chrimson channels open when exposed to red light, allowing cation influx and depolarizing the neuron. Elevated intracellular calcium increases GCaMP fluorescence and neuron brightness in the microscope image. (b) Neural responses in AWA for 15 animals illuminated by the blue low-cost LED system and stimulated with a 10 s pulse of the red low-cost LED system. For comparison, the same animals were imaged using the commercial system for both blue and red exposure. Heatmap shows increase in neural fluorescence relative to baseline as dF/F_0_ where F is pixel value integrated over the neuron soma. Baseline F_0_ is calculated from 4 s prior to stimulation. (c) Comparison of low-cost and commercial systems. Line represents mean; shading represents standard error, n = 15 animals. (d) Comparison of baseline signal, signal noise, and peak AWA response in low-cost and commercial systems. Statistical analysis by paired *t*-test, **p < 0.001, ns – no significance. (e) Peak AWA neural responses for individual animals illuminated by both sources. (For interpretation of the references to colour in this figure legend, the reader is referred to the web version of this article.)

Animals expressing the calcium sensor GCaMP and light-activated cation channel Chrimson (strain NZ1091) were grown overnight on OP50 *E. coli* lawns containing 50 µM all-trans retinal (ATR), a cofactor required for Chrimson function. Animals were loaded into a microfluidic device with 1 mM tetramisole hydrochloride in S. basal buffer to induce paralysis over 30 min (Fig. 11a). The commercial system (Mightex 50 W blue and 38 W red, with 2 m liquid LGs) was first connected to the microscope for neural recordings, followed by the optimized low-cost 3 W LED systems (with 5 mm spacer at the microscope LG launch), allowing the same animals to be imaged with both systems about ∼ 20 min apart. Neural responses were recorded by 10 ms blue (470 nm) GCaMP excitation pulses at 10 fps and stimulated by 10 s continuous red (625 nm) light from 5 – 15 s during the 30 s long recording (Fig. 11b). Image stacks were analyzed for integrated neural fluorescence (*F*) using NeuroTracker software [26]. The software calculates pre-stimulus fluorescence (*F_0_*) and the relative increase in fluorescence (*ΔF/F_0_*) above baseline which represents neural activation (Fig. 11b). Average normalized neural responses were nearly identical between the commercial and low-cost LED systems (Fig. 11c). While the brighter commercial illumination resulted in a two-fold higher baseline signal, it did not significantly reduce neural signal noise compared with the low-cost LED system (2.4 ± 0.7 % (s.d.) vs. 2.5 ± 0.5 %, p = 0.7) (Fig. 11d). Further, average peak neural responses were quantitatively equivalent (126 ± 39 % (s.d.) vs. 136 ± 41 %, p = 0.12) and individual animal peak responses were highly correlated between the two illumination systems (Pearson’s r = 0.86, p < 10^-4^; Fig. 11e).

Overall, the low-cost 3 W LED illumination system with plastic light guide achieved comparable optogenetic activation and neural imaging to the high power, high-cost systems with 38 W and 50 W illumination sources and a liquid light guide. The low-cost 3 W system provided an intensity output comparable to commercial 7 W and 15 W systems, with gains in efficiency permitting passive cooling instead of active fan cooling.

### 7.6 Summary

Solid-state LED illumination systems for microscopy are generally expensive, but many applications are well served by low-cost, bright, customizable LED systems. Here, a broadly available 3 W LED module, simple controller circuit, and plastic light guide successfully duplicated optogenetic neural imaging experiments while costing about 20-fold less than commercial alternatives. By keeping the LED light beam within the width of the light guide, no collecting or collimating lenses were needed. 3D printed parts allowed for simple alignment of the light guide to the LED, and a small plastic ring surrounding the LED increased intensity by about 35 % by reflecting off-axis light into the LG. Output power was nearly 300 mW at the light guide exit and 40 – 60 mW at the sample plane of our microscope, with high flatfield uniformity, comparable to 7 W to 15 W commercial systems. Low electrical power use (5 W) and small physical size enable its use in resource-limited environments and compact, battery-powered imaging devices.

### 7.7 Limitations and potential improvements

The low-cost LED system demonstrates a significant enhancement in radiant flux using easily available white plastic for reflection around the LED, but also room for further improvement. Other materials with increased reflectivity could increase light output by an additional 50 % (see section 7.2). For example, increased reflectance could be achieved by specialty plastic filaments such as Reflect-o-Lay (3DPrima.com, item 22157), reflective paint, or a polished metal ring incorporated into the mount. Additionally, minor modification of 3D printed parts would accommodate larger diameter light guides and larger diameter LEDs to transmit more light.

While the inexpensive plastic materials performed well in our tests, degradation of the PETG spacer ferrule and PMMA LG is possible, especially with high-intensity blue and UV LEDs. The 3 W blue LED exceeded the transition temperatures of both plastics after one minute of continuous exposure. UV wavelengths (<420 nm) transmit poorly through PMMA, and UV-B/C exposure (<320 nm) degrades PMMA mechanically and optically, causing yellowing, increased light scattering, and reduced visible light transmission [27]. Therefore, a liquid LG and high-temperature filament (e.g., polycarbonate) are recommended for continuous blue illumination and UV exposure.

Many bioimaging applications require multiple excitation wavelengths delivered though a single LG. For example, the Angstrom system [8] combines multiple LEDs and LGs into one LG output, but some light intensity is lost. The primary advantage of a single LED per LG is that it allows the LG to be brought very close to the LED for high intensity output without relay lenses or mirrors. High-power multicolor LEDs, such as the Cree XML RGBW Star, contain four LED emitters (red, green, blue, white) in a single housing, with wavelength peaks at 620, 520, and 450 nm. Alternately, broadband white LEDs can be filtered at the microscope to provide many excitation colors, although it should be noted that “white” emitters typically have a violet peak at 440 nm directly from the LED and a broad green–red (500 – 650 nm) band from the phosphor coating, but little intensity in the 470 – 490 nm excitation range for common green fluorophores such as fluorescein and green fluorescent protein (GFP).

Over half of the cost of the present design comes from the machined aluminum case and heatsink, which could be substantially lowered using inexpensive extruded aluminum heatsinks. The inclusion of a small fan could further reduce operational temperatures, and multiple LED/LG assemblies can be incorporated into a case sharing a single electrical power input.

Depending on the use case, any of these modifications can likely be made with minimal added cost. It is recommended to verify LED temperatures, output intensities, flatfield illumination, and long-term material stability following any changes, as presented here, and we encourage open sharing of modified designs.

## Ethics statements

No human or animal subjects were used in this study.

## CRediT authorship contribution statement

**Fox Avery:** Writing – review & editing, Writing – original draft, Visualization, Validation, Methodology, Investigation, Data curation, Conceptualization. **Thomas Geer:** Writing – review & editing, Resources, Conceptualization. **Dirk Albrecht:** Writing – review & editing, Writing – original draft, Supervision, Resources, Methodology, Funding acquisition, Conceptualization.

## Declaration of competing interest

The authors declare the following financial interests/personal relationships which may be considered as potential competing interests: Thomas Geer is affiliated with Nobska Imaging, Inc., a provider of scientific imaging solutions which includes sales of commercial microscopy illumination systems.
