# Supplementary material for: Lensless light guide-coupled LED illumination for low-cost microscopy
Source: HardwareX. 2025 Nov 24;24:e00725. doi: 10.1016/j.ohx.2025.e00725 (PMC12744834; doi:10.1016/j.ohx.2025.e00725)

Lensless light guide-coupled LED illumination for low-cost microscopy

Design files summary

Design file name	File type	Open source license	Location of the file
P1_Optics_Holder.stl<br>P2_Spacer_Ferrule.stl<br>P3_Light_Guide_Ferrule.stl<br>P4_Cable_Guide.stl	CAD files	CC-BY	Thingiverse<br>https://www.thingiverse.com/thing:6971030

These design files contain all required parts (P1 – P3) and optional cable guides (P4) in the recommended printing orientations. Files can be imported to any 3D printing slicer software to generate Gcode for any 3D printer.

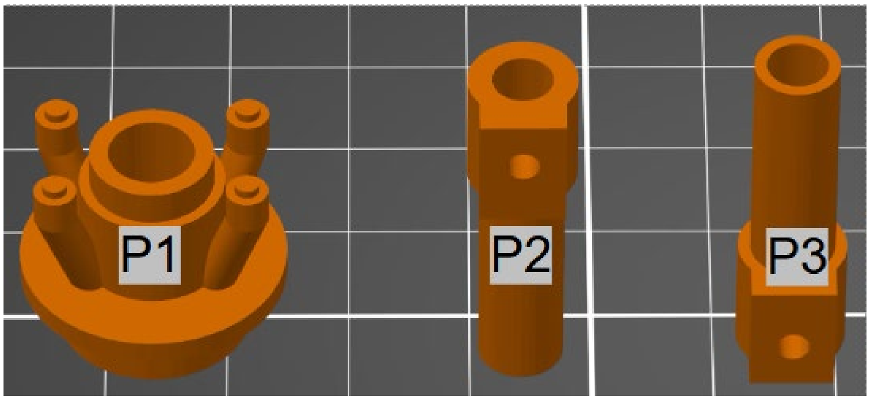

Supplement: Supplementary Data 1 [file mmc1.pdf]
